# How Is Ethical Leadership Linked to Subordinate Taking Charge? A Moderated Mediation Model of Social Exchange and Power Distance

**DOI:** 10.3389/fpsyg.2020.00315

**Published:** 2020-03-20

**Authors:** Qiao Wang, Xiaohu Zhou, Jiani Bao, Xueyan Zhang, Wei Ju

**Affiliations:** ^1^School of Economics and Management, Nanjing University of Science and Technology, Nanjing, China; ^2^School of Business and Administration, Nanjing University of Finance and Economics, Nanjing, China

**Keywords:** ethical leadership, social exchange, power distance, subordinate taking charge, moderated mediation model

## Abstract

Extant literature has suggested that leadership styles have a significant impact on subordinate taking charge. However, the effect of ethical leadership on subordinate taking charge is still insufficiently explored. Drawing on social exchange theory, we developed a moderated mediation model in which social exchange was theorized as a mediating mechanism underlining why subordinates feel motivated to take charge with the supervision of ethical leadership. Moreover, power distance was supposed to be a relevant boundary condition to moderate such a relationship. Two hundred thirty-nine independent leader–subordinate dyads in China were used to test the model. Results showed that subordinates’ social exchange mediates the relationship between ethical leadership and subordinate taking charge, and such a relationship was found to be stronger among subordinates who had lower levels of power distance rather than higher levels. Theoretical and practical implications concerning enhancement of subordinate taking charge in organizations where ethical leaderships exist are discussed.

## Introduction

Taking charge has emerged as a major means of enhancing management effectiveness in the environment of rapidly changing, high-competition, and highly uncertain business (Crant, 2000; Grant and Ashford, 2008), which is an important form of proactive behavior (Morrison and Phelps, 1999; Parker and Collins, 2010) and prosocial behavior (Grant et al., 2009; Lee, 2016). Taking charge has been defined as “voluntary and constructive efforts, by individual subordinates, to effect organizationally functional change with respect to how work is executed within the contexts of their jobs, work units, or organizations”(Morrison and Phelps, 1999). Previous studies have recognized various precursors of taking charge from three aspects: individual factors, such as self-efficacy (Morrison and Phelps, 1999) and proactive personality (Fuller et al., 2012); contextual factors, such as working group norms (Morrison and Phelps, 1999), organizational support (Burnett et al., 2015), and sustainable human resource management (Li et al., 2019); and leadership factors, such as transformational leadership (Ning et al., 2013), empowering leadership (Li et al., 2015), self-sacrificial leadership (Li et al., 2016), and leader humility (Zhang and Liu, 2019). Ethical leadership is an important leadership style, which has received extensive attention from academic scholars and practitioners of modern organizational management. But the relation between ethical leadership and taking charge has not been clearly researched. Therefore, it is of significant interest to explore how ethical leadership influences subordinate taking charge.

Ethical leadership has personal traits of honesty, altruism, and trustworthiness (Brown and Treviño, 2006; Kalshoven et al., 2011) and allows employees to take part in decision making and encourages subordinates to voice their ideas and methods for improving work procedures (Walumbwa and John, 2009). Scholars have shown that ethical leadership has a positive influence on a lot of subordinate outcomes, such as voice (Zhu et al., 2015; Hu et al., 2018), organizational citizenship behavior (Shareef and Atan, 2019), followers’ creativity (Javed et al., 2018), and job performance (Mo and Shi, 2016). Although the relationship between ethical leadership and taking charge has been explored by Lee (2016), who explored the effect of ethical leadership on taking charge and used soldiers of the South Korean Navy instead of employees in the business workplace. According to Wong et al. (2003), the characteristic of business organizations is different from military organizations, so whether the relationship between ethical leadership and taking charge verified in the military organization also exists in business organizations needs to be verified. Thus, it still contained crucial gaps over underlying processes that explain how ethical leadership affects taking charge in the business workplace.

To illuminate whether and how ethical leadership associates with taking charge in a real work context, we employ a social exchange perspective (Blau, 1964). Social exchange theory is a relevant theoretical framework to explore whether and how subordinates are motivated to engage in taking charge under the influence of ethical leadership. Scholars have indicated that social exchange is a potential mediated mechanism for ethical leadership to influence subordinates’ work-related behavior (Brown and Treviño, 2006; Garba et al., 2017). According to social exchange theory (Blau, 1964), subordinates perceive a high-quality social exchange relationship with ethical leadership; social exchange would incline us to produce feelings of personal gratitude, obligation, and trust (Blau, 1964), which motivate subordinates to pay back with positive attitudes and beneficial working behaviors (Kacmar et al., 2011). Thus, we put forward that social exchange mediates the relationship between ethical leadership and subordinate taking charge.

**FIGURE 1:**
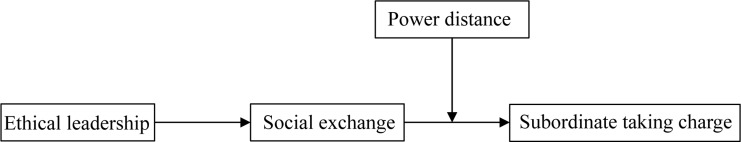
Hypothesized research model.

Although recent researches have indicated that ethical leadership can influence subordinates’ work-related behaviors (Garba et al., 2017; Walumbwa et al., 2017; Belschak et al., 2018), we still do not know whether subordinates with different cultural values react to ethical leaderships differently (Zhu et al., 2015). Brown and Mitchell (2010) have shown that differences in subordinates’ cultural values (such as power distance) could influence the effect of ethical leadership on subordinates’ work-related outcomes. In addition, it has indicated in recent research that though within the same culture, there are obvious differences in every individual’s cultural value (Farh et al., 2007; Kirkman et al., 2009; Lian et al., 2012). Therefore, extending our understanding of how to manage different human capital that holds diverse cultural values effectively is important. Power distance is not merely one of the most relevant cultural values in China (Farh et al., 2007; Lin et al., 2018) but also the most relevant to the study of social exchange (Hui et al., 2004; Lin et al., 2018). Power distance represents exactly individuals’ fundamental beliefs and values of power, which shape the effect of social exchange on individuals’ perceptions, attitudes, and behaviors. Therefore, our study argues that power distance has an important role in determining the relationships between ethical leadership, social exchange, and subordinate taking charge. The complete research model is shown in Figure 1.

Our research has contributed to existing literature in three dimensions. First, our study expands the literature of taking charge and provides and tests that ethical leadership positively impacts subordinate taking charge in the real work context. Second, drawing on social exchange theory, we identify social exchange as a critical mediating process through which ethical leadership affects subordinate taking charge. Meanwhile, we empirically test the recommendations of Brown and Treviño (2006) to use social exchange as a critical process for ethical leadership to influence subordinate outcomes. Third, we explore the contextual boundary condition of the effect of ethical leadership on subordinate taking charge. Specifically, our study explores the way to theorize and exemplify how social exchange interacts with power distance to influence subordinate taking charge. The interplay between social exchange and power distance in promoting the processes of subordinate taking charge can offer theoretical and practical insights.

### Theory and Hypothesis

#### Ethical Leadership and Subordinate Taking Charge

Ethical leadership refers to “the demonstration of normatively appropriate conduct through personal actions and interpersonal relationships and the promotion of such conduct to followers through two-way communication, reinforcement, and decision-making” (Brown et al., 2005). And ethical leadership includes two dimensions: moral person and moral manager (Treviño et al., 2003). The moral personal dimensions include the personal traits, such as honesty, credibility, altruism, and trustworthiness (Kalshoven et al., 2011). The moral manager dimension includes the proactive influence of leaders, such as discussing ethical issues with subordinates, showing concern and respect for subordinates, and using discipline and reward to make subordinates accountable for ethical behavior (Brown and Treviño, 2006). Ethical leadership has a beneficial role in fostering subordinate taking charge from three aspects: role model, obligation, and risk.

Firstly, ethical leadership can influence subordinates as a role model (Zhu et al., 2016); leaders are seen as responsible and trustful and as having initiative to improve work-related procedures (Brown et al., 2005), so subordinates may imitate them (Cheng et al., 2019).

Secondly, ethical leadership shows trust, care, and authority to subordinates (Walumbwa et al., 2011), which would help develop a high-quality relationship with between leaders and their subordinates. Therefore, the subordinates would develop a strong sense of responsibility, gratitude, and trustworthiness and perceive an obligation to reciprocate by proactive work-related behaviors and even act beyond their job responsibilities (Mo and Shi, 2017; Shareef and Atan, 2019). For example, when they find that they can improve the efficiency of the organization, they would conduct proactive behavior for the benefit of the organization.

Thirdly, ethical leadership could promote subordinates taking charge by avoiding the risk of retaliation (Cheng et al., 2019). There is a popular Chinese saying that goes “one who sticks his neck out gets hit first,” which means subordinates would think that taking charge has potential risk (Morrison and Phelps, 1999; Li et al., 2016). However, ethical leaders care about “how we can do correctly” and “what is right”; they reckon taking charge is helpful for organizations and will support, appreciate, and even reward subordinates (Brown et al., 2005). This attitude will affect subordinates, who will think that taking charge is supported by superiors. Such protection measurements are expected to minimize the risk of taking charge and encourage it. Therefore, the following assumptions are proposed:

Hypothesis 1: Ethical leadership is positively related to subordinate taking charge.

#### Ethical Leadership and Social Exchange

According to Blau (1964), social exchange does not require a broad obligation to return in the future; it must be decided by the person who makes the return themselves, not by negotiating the real condition of the return. Shore et al. (2006) argued that social exchange generates trust, provides broad investment, emphasizes socio-emotional input, and focuses on long-term orientation, which distinguish it from economic exchange. Social exchange exists widely between subordinates and different groups, such as among employees and organizations that employ them, colleagues, and their direct leaders (Tekleab et al., 2005; Lavelle et al., 2007; Wang et al., 2018). When social exchange occurs between subordinates and their direct leaders, subordinates’ treatment affects their perception of social exchange (Wayne et al., 1997; Hinkin and Schriesheim, 2015; Carnevale et al., 2019; Chen et al., 2019). For example, ethical leadership treats subordinates’ honestly, credibly, and trustworthily, which affects the social exchange relationship between subordinates and their leaders (Shore et al., 2006).

Based on previous researches (Song et al., 2009; Wang et al., 2018; Chen et al., 2019), our study proposes that ethical leadership affects the subordinates’ perception of social exchange, which reflect social exchange from three aspects, such as trust, socio-emotional input, and long-term orientation. First, precious researches have shown that when subordinates receive fairness, sincere treatment, and care from their direct leaders, they would trust their direct leaders more (Hinkin and Schriesheim, 2015). In social exchange between subordinates and their direct leaders, mutual trust is seen as the basis of the social exchange relationship (Shore et al., 2006), because the trust expressed firstly usually gets equal trust in return (Mcknight et al., 1998). Second, Brown and Treviño (2006) argue that ethical leadership has traits of honesty, credibility, and altruism. They consciously take the initiative to demonstrate ethical behavior, such as respecting subordinates, caring for subordinates, and making fair decisions, and they use incentives to motivate subordinates to take responsibility for ethical behavior (Brown and Treviño, 2006; Byun et al., 2018). Subordinates would understand these behaviors of ethical leadership by caring about their social needs, emotional needs, and socio-emotional input (Shore et al., 2006; Song et al., 2009). Therefore, subordinates are more likely to realize that they have a social exchange relationship with their leaders (Brown and Treviño, 2006). Finally, Blau (1964) argued that the benefits involved in social exchange are not priced for a single quantitative exchange, which means that social exchange can generate a lasting social pattern (Cropanzano and Mitchell, 2005). Specifically, social exchange involves the repeated exchange of interest, which creates a sense of obligation to return (Shore, 2007). Therefore, we propose the following:

Hypothesis 2: Ethical leadership is positively related to social exchange.

#### Social Exchange and Subordinate Taking Charge

Taking charge is a subordinate’s discretionary behavior that usually conducts constructive change and could bring about positive effects on the organization’s efficiency (Morrison and Phelps, 1999). Unlike other proactive behaviors, such as individual innovation, voice, and organizational citizenship behaviors (Bies, 1988; Scott and Bruce, 1994; Walumbwa et al., 2012), taking charge has characteristics of being change oriented, risky, and proactive (Morrison and Phelps, 1999; Mcallister et al., 2007). Therefore, whether subordinates take part in taking charge relies on the form of leadership and managerial practices in organizations (Crant, 2000; Ning et al., 2013; Li et al., 2015; Xu et al., 2018).

According to social exchange theory (Blau, 1964), when subordinates receive trust and socio-emotional input from and form long-term relationships with their leaders, subordinates would perceive a high-quality relationship of social exchange and believe that there is an obligation to return beneficial work-related behaviors (Brown and Treviño, 2006; Hansen et al., 2013; Garba et al., 2017), such as taking charge. Precious researches have extensively examined leadership–member exchange (Kim et al., 2015; Dhar, 2016), supervisor support (Murry et al., 2001; Byrne et al., 2012), and trust in the leader (Lee, 2016; Javed et al., 2018) to reflect the concept of social exchange between subordinates and their direct leaders. Among them, leadership–member exchange depends on the relationship quality between leaders and their subordinates (Uhlbien, 1995). Subordinates with high-quality leadership–member exchanges can get more work-related resources (Graen and Scandura, 1987) and accreditation (Graen and Cashman, 1975). At the same time, they would accept more challenging tasks (Liden and Graen, 1980), which would make them more willing to take risks associated with work (Graen and Cashman, 1975). Perceived leadership support means that the leader values the contributions of subordinates and cares about their well-being (García-Cabrera et al., 2018; Lucia-Casademunt et al., 2018). Therefore, if leaders support subordinates and provide sufficient resources, subordinate taking charge would be enhanced (Burnett et al., 2015; Zhen and Xu, 2017; Nathan et al., 2018). Besides, Kim et al. (2015) also have shown that leadership–member exchange was positively related to subordinate taking charge. Furthermore, social exchange tends to engender feelings of individual obligation which would incline subordinates to go beyond the call of work-related duty (Brown and Treviño, 2006). Therefore, we propose the third hypothesis:

Hypothesis 3: Social exchange is positively related to subordinate taking charge.

#### Mediating Role of Social Exchange

Integrating the above argument in Hypotheses 1, 2, and 3, we further anticipate that ethical leadership influences subordinate taking charge indirectly via social exchange. Specifically, Lee (2016) has suggested that ethical leadership could promote taking charge, but this study lacked empirical evidence that can support this conclusion in the business workplace. Social exchange is considered a critical precursor to work-related behavior (Settoon et al., 1996; Brown and Treviño, 2006; Lavelle et al., 2007), and ethical leadership is a crucial factor to foster social exchange (Garba et al., 2017). So it is logical that ethical leadership would promote subordinates’ taking charge when leaders encourage the development of social exchange (Brown and Treviño, 2006). Therefore, our study proposes that ethical leadership is an important precursor to social exchange, which would be associated with social exchange and would enhance subordinate taking charge in turn. Thus, we hypothesize the following:

Hypothesis 4: Social exchange mediates the relationship between ethical leadership and subordinate taking charge.

#### Moderating Role of Power Distance

Previous researches have shown that there were significant differences among different individuals with equal cultural value (Clugston et al., 2000; Kirkman and Shapiro, 2001; Sue-Chan and Ong, 2002; Kirkman et al., 2006). Power distance is one of the four dimensions of cultural values (Hofstede, 1980); researches also have shown that power distance may influence subordinates’ social exchange (Hui et al., 2004; Lin et al., 2018). Based on existing researches, our study proposes that power distance would moderate the relationship between social exchange and subordinate taking charge.

Power distance at the individual level has been defined as “the extent to which an individual accepts the unequal distribution of power in institutions and organizations” (Clugston et al., 2000; Kirkman et al., 2009). Specifically, individuals with high power distance would accept unbalanced distribution of power, treat unfairness as justified, and be less sensitive to fairness or inequality. Individuals with low power distance would be more concerned about the balanced distribution of power, unable to accept unfair treatment, and more sensitive to fairness or inequality (Cong et al., 2013). Farh et al. (2007) indicated that the reaction of subordinates with high power distance to leadership behavior does not depend on how leaders treat them or that whether leaders treat subordinates in a fair manner does not have a significant impact on the psychology of subordinates with high power distance. But for subordinates with low power distances, how leaders treat them would affect them to a greater extent. In other words, whether the leaders fairly treat subordinates would have a more significant impact on the psychology of subordinates with low power distance.

Based on the above logic, subordinates with high power distances are not sensitive to whether they are treated fairly and do not care whether the distribution of power is fair. Regardless of the level of social exchange between supervisors and subordinates (high or low), subordinates are more likely to show understanding and acceptance. Therefore, it has no significant impact on subordinate taking charge who with a high level of power distance, which is similar to previous views that the attitudes and behaviors of subordinates have a lower explanation to individuals through social exchange theory (Lee et al., 2000; Brockner et al., 2001; Schaubroeck and Aryee, 2002). Subordinates with low power distance are more sensitive to fairness or equality. Therefore, when the quality of social exchange between supervisors and subordinates is high, such as more trust, socio-emotional input, and long-term orientation, subordinates with low power distance would meet their psychological needs, which would promote subordinates taking charge. On the contrary, when the quality of social exchange between supervisors and subordinates is low, subordinates could not meet their psychological needs, so subordinate taking charge would be suppressed. Thus, we propose the following moderation hypothesis:

Hypothesis 5: The relationship between social exchange and subordinate taking charge will be stronger when subordinates have a low rather than a high level of power distance.

Beyond the moderating effects of power distance on the relationship between social exchange and subordinate taking charge, it is logical to predict that power distance would affect the strength of the indirect relationship between ethical leadership and subordinate taking charge in some conditions. It is expected that this indirect relationship could be enhanced by low power distance, especially when strengthening the mediating role of social exchange between ethical leadership and subordinate taking charge. Hence, subordinates who have low power distance are inclined to stimulate their beneficial work-related behaviors and to take charge under ethical leadership’s supervision that facilitates subordinates’ social exchange. Therefore, we suppose a moderated mediation model to illustrate the influence of ethical leadership on subordinate taking charge; we also assume a strong relationship between ethical leadership and subordinate taking charge when the power distance of the subordinate is low. Therefore, the last hypothesis is as follows:

Hypothesis 6: Power distance will moderate the mediated effect of ethical leadership on subordinate taking charge via social exchange such that the indirect relationship will be stronger when there is a low rather than high level of power distance.

## Materials and Methods

### Participants and Procedures

Our data were collected from 10 Chinese companies from three industries, namely, telecommunications, biotechnology, and real estate. All companies selected were from the Guangdong, Jiangsu, and Hubei provinces. We surveyed each company following procedures. Firstly, we contacted the relevant executive manager of the company, and then we asked human resources managers to provide five or more work teams randomly. The appointed teams all came from official departments and had a long-term cooperative relationship within the organization. Therefore, each team only had one official leader, and the members were familiar with each other; they also contacted each other frequently in the workplace. Second, before implementing the survey, we informed the participants that they need volunteer to join in this survey and that their participation is valuable; we also told them that any information related to these data would be used for academic aim only and that this survey was anonymous. All members within the appointed teams were required to fill in questionnaires to report their personal demographic information and to assess ethical leadership, social exchange, and power distance. Moreover, they needed to fill in the last four digits of their working mobile phone number. Thirdly, we sent questionnaires to team leaders, and they needed to report their tenure in their current position and assess the taking charge of subordinates; the last four digits of the evaluated subordinates’ working mobile phone number were also required. The process of data collection satisfies the ethical standard. Before collecting data, the study consulted the ethics committee of the School of Economics and Management of Nanjing University of Science and Technology, which approved the study. According to the study design, the research has not violated any laws, regulations, and common ethics.

In total, we received 260 leader–subordinate dyads from 52 work teams. After matching the leader and member questionnaires, we eliminated 21 subordinate–supervisor dyads from the collection because the last four digits of the mobile phone number did not match. The final sample consisted of 239 follower questionnaires and 239 leader questionnaires, with an average response rate of 91.92%. Most respondents were male (54.39%), were below 30 years of age (80.33%), and had a bachelor’s degree (42.68%). Of these respondents, 32.22% had a team tenure of between 1 and 2 years, and 34.73% had an income between 5,001 and 10,000 RMB.

### Measures

Because all the measuring instruments we used were originally written in English, we translated them into Chinese by using Brislin’s (1980) back-translation procedure. Subordinates were asked to assess ethical leadership, power distance, and social exchange, while leaders rated their subordinates’ taking charge. All responses were measured with the six-point Likert scale (1 = strongly disagree, 6 = strongly agree).

#### Ethics Statement

Subordinate participants used Brown et al.’s (2005) 10-item measures to assess ethical leadership. One example item is “Define success not only in terms of results, but also in terms of how you get the results.” The Cronbach alpha value was 0.87.

#### Social Exchange

Subordinates used the eight-item scale of Rupp and Cropanzano (2002) to indicate the level of social exchange they experienced. One example is “My direct manager made a significant investment in me.” The Cronbach alpha value was 0.85.

#### Power Distance

Subordinate participants used the eight-item scale of Dorfman and Howell (1988) to assess the power distance they experienced in the workplace. One example is that “Managers should make most decisions without consulting their subordinates.” The Cronbach alpha value was 0.84.

#### Subordinate Taking Charge

Superiors used the 10-item scale proposed by Morrison and Phelps (1999) to evaluate the subordinate taking charge. One example is “Subordinate who often tries to correct a faulty procedure or practice.” The Cronbach alpha value was 0.86.

#### Control Variables

Because of the potential influence of individual demographics, our study set gender, age, education level, and team tenure as control variables. Li et al. (2016) set gender and age as control variables, which had been found to be related to subordinate taking charge because it is associated with uncertainty and risk. Similarly, Li et al. (2015) showed that education level related to subordinate taking charge: subordinates who have a high level of education might have more knowledge accumulation and know better how to implement these behaviors. Moreover, Kim et al. (2015) found that subordinate behaviors were influenced by team tenure, which meant that freshmen in the workplace were less engaged in taking charge. Additionally, we also required subordinates to report their incomes, which might be an antecedent of subordinate taking charge.

## Results

### Confirmatory Factor Analysis

For the sake of examining the data, we executed a confirmatory factor analysis (CFA) to assess the variables’ validity. Table 1 shows the test results for the competing CFA model. The outcomes of the hypothesized four-factor measurement model display a better model fit (χ^2^ = 818.83, *df* = 521, IFI = 0.91, TLI = 0.90, TLI = 0.91, RMSEA = 0.05), which is compared to the alternative measurement model. These results provide the basis for the discriminant and convergence of our measure.

**TABLE 1:** Results of confirmatory factor analysis.

Variable	χ^2^	*df*	χ^2^/*df*	IFI	TLI	CFI	RMSEA
Four-factor model	818.83	521	1.57	0.91	0.90	0.91	0.05
Three-factor model	1,124.47	524	2.15	0.83	0.81	0.83	0.07
Two-factor model	1,631.85	526	3.10	0.68	0.66	0.68	0.09
One-factor model	2,355.80	527	4.47	0.47	0.43	0.47	0.12

*Four-factor model: ethical leadership, social exchange, power distance, and taking charge. Three-factor model: combining ethical leadership and social exchange. Two-factor model: combining ethical leadership, social exchange, and power distance. One-factor model: combining all four constructs into one factor.*

### Common Method Variance

Although our data were collected from the subordinates and their leaders to mitigate the effect of common method variance (CMV) (Podsakoff et al., 2012), the independent variable (i.e., ethical leadership) and mediating variable (i.e., social exchange) were derived from the self-report of subordinates, which may cause problems of CMV. We adopted two methods to test whether CMV exists in this research. Firstly, we use the single-factor test method of Hair et al. (1998) to test CMV; the results show that the first factor can explain 20.09% of variances, which is far below 40%. It shows that the CMV of the data is not significant and will not affect the reliability of the research conclusion. Additionally, similar to Podsakoff et al. (2003) and Liang et al. (2007), our research examined CMV by the partial least squares (PLSs) model, whose indicators included all the indicators of principal constructs and which calculated each variance of indicators explained by the principal construct and this method. It is shown in Table 2 that the substantively explained variance of the indicators is 0.38 in average and that the average method-based variance is 0.07. The ratio of substantive variance to method variance is about 5:1. Additionally, most method factor loadings are not significant. Thus, the results of PLS show that the CMV of the data is not significant in our research.

**TABLE 2:** Common method bias analysis.

Construct	Indicator	Substantive factor loading (*R*1)	*R*1^2^	Method factor loading (*R*2)	*R*2^2^
Ethical leadership	EL1	0.49**	0.24	0.24	0.06
	EL2	0.76**	0.58	0.27	0.07
	EL3	0.79**	0.62	0.27	0.07
	EL4	0.76**	0.57	0.28*	0.08
	EL5	0.73**	0.53	0.27	0.07
	EL6	0.76**	0.57	0.26	0.07
	EL7	0.67**	0.44	0.26	0.07
	EL8	0.40**	0.16	0.26	0.07
	EL9	0.33**	0.11	0.28	0.08
	EL10	0.23**	0.05	0.21	0.05
Social exchange	SC1	0.48**	0.23	0.24	0.06
	SC2	0.70**	0.50	0.27**	0.07
	SC3	0.51**	0.26	0.25	0.06
	SC4	0.82**	0.67	0.26	0.07
	SC5	0.73**	0.54	0.23	0.05
	SC6	0.59**	0.35	0.28	0.08
	SC7	0.41**	0.16	0.25	0.06
	SC8	0.48**	0.23	0.28	0.08
Power distance	PD1	0.56**	0.31	0.21*	0.04
	PD2	0.43**	0.18	0.23	0.05
	PD3	0.68**	0.46	0.20	0.04
	PD4	0.79**	0.62	0.19	0.04
	PD5	0.67**	0.45	0.20	0.04
	PD6	0.81**	0.65	0.20*	0.04
Subordinate taking charge	STC1	0.13**	0.02	0.28	0.08
	STC2	0.38**	0.14	0.31*	0.10
	STC3	0.67**	0.44	0.28	0.08
	STC4	0.63**	0.40	0.25	0.06
	STC5	0.66**	0.43	0.27	0.07
	STC6	0.71**	0.50	0.26	0.07
	STC7	0.66**	0.44	0.28	0.08
	STC8	0.55**	0.30	0.29**	0.08
	STC9	0.61**	0.38	0.29	0.08
	STC10	0.50**	0.25	0.30	0.09
**Average**		**0.59**	**0.38**	**0.26**	**0.07**

* p < 0.05, ** p < 0.01.

### Descriptive Statistics

Table 3 indicates the descriptive statistics and correlations among various research variables. As expected, the core study variables were related to each other. Specifically, ethical leadership was positively related to social exchange (*r* = 0.57, *p* < 0.01) and subordinate taking charge (*r* = 0.17, *p* < 0.01). Social exchange was positively correlated with subordinate taking charge (*r* = 0.30, *p* < 0.01). Besides, power distance was negatively associated with social exchange (*r* = −0.26, *p* < 0.01), but it was positively associated with subordinate taking charge (*r* = 0.43, *p* < 0.01).

**TABLE 3:** Means, standard deviations, and correlations.

Variable	*M*	*SD*	1	2	3	4	5	6	7	8	9
(1) Gender	1.46	0.50	1.00								
(2) Age	1.26	0.59	–0.11	1.00							
(3) Education level	2.96	0.92	0.02	−0.18**	1.00						
(4) Team tenure	2.13	1.08	–0.05	0.50**	–0.09	1.00					
(0. Income	2.29	1.09	–0.00	0.00	0.36**	0.17**	1.00				
(6) Ethical leadership	4.62	0.71	–0.01	–0.07	0.13	−0.13*	–0.04	1.00			
(7) Social exchange	4.37	0.74	–0.05	–0.02	0.11	–0.06	–0.03	0.57**	1.00		
(8) Power distance	3.59	0.99	–0.00	0.11	−0.17**	0.12	–0.03	−0.37**	−0.26**	1.00	
(9) Taking charge	4.14	0.67	0.04	0.10	–0.11	0.12	–0.06	0.17**	0.30**	0.43**	1.00

Gender: 1 = “male,” 2 = “female.” Age: 1 = “30 years and below,” 2 = “31 to 40 years,” 3 = “41 to 50 years,” 4 = “51 and above 51 years.” Education level: 1 = “high school and below,” 2 = “junior college,” 3 = “bachelor degree,” 4 = “master degree and above.” Team tenure: 1 = “below 1 year,” 2 = “1 to 2 years,” 3 = “3 to 5 years,” 4 = “6 to 10 years,” 5 = “11 years and above.” Income: 1 = “below 5000 RMB,” 2 = “5,001 to 10,000 RMB,” 3 = “10,001 to 15,000 RMB,” 4 = 15,000 to 20,000 RMB,” 5 = “above 20,001 RMB.” N = 239; * p < 0.05, ** *p* < 0.01 (two-tailed test).

### Hypothesis Testing

Our study used a hierarchical multiple regression technique to analyze Hypotheses 1, 2, 3, and 4, in which we added a dependent variable (subordinate taking charge), control variables, an independent variable (ethical leadership), a mediator variable (social exchange), a moderator variable (power distance), and an interaction variable (social exchange multiplied by power distance) on a successive series of steps.

Hypothesis 1 assumes a positive relationship between ethical leadership and subordinate taking charge. The results of Model 4 in Table 4 show that ethical leadership was positively related to subordinate taking charge (β = 0.20, *p* < 0.001). Therefore, Hypothesis 1 is supported.

**TABLE 4:** Hierarchical multiple regression analysis.

	Social exchange		Subordinate taking charge					
	Model 1	Model 2	Model 3	Model 4	Model 5	Model 6	Mode 7	Model 8
**Control variable**								
Gender	–0.08	–0.07	0.06	0.07	0.09	0.07	0.09	0.06
Age	0.03	0.02	0.05	0.05	0.04	0.01	0.01	0.01
Education level	0.02	0.04	–0.06	–0.08	–0.09	–0.04	–0.05	–0.04
Team tenure	–0.03	0.01	0.07	0.08	0.08	0.05	0.06	0.06
Income	–0.05	–0.02	–0.03	–0.02	–0.01	–0.02	–0.01	–0.01
**Main variables**								
Ethical leadership		0.59***		0.20**			0.20**	0.22***
Social exchange					0.30***	0.41***	0.31***	0.30***
Power distance						0.32***	0.38***	0.36***
Moderation effect								
**Social exchange × Power distance**						−0.21***		−0.22***
Δ*F*	1.60	105.16***	1.43	10.17**	27.45***	22.17***	31.02***	30.29***
*R*^2^	0.02	0.33	0.03	0.07	0.11	0.44	0.41	0.47
Δ*R*^2^	0.02	0.31	0.03	0.04	0.10	0.05	0.08	0.14

*P < 0.05, **P < 0.01, ***P < 0.001.

Hypothesis 2 assumes a positive relationship between ethical leadership and social exchange. The results of Model 2 in Table 3 show that ethical leadership was positively related to social exchange (β = 0.59, *p* < 0.001). Thus, Hypothesis 2 is supported.

Hypothesis 3 assumes a positive relationship between social exchange and subordinate taking charge. The results of Model 5 in Table 4 show that social exchange was positively related to subordinate taking charge (β = 0.30, *p* < 0.001). Thus, Hypothesis 3 is supported.

Hypothesis 4 proposed that social exchange mediates the relationship between ethical leadership and subordinate taking charge. To test the mediation effect, we used bias-corrected bootstrapping techniques by the Hayes (2013) PROCESS macro. The results in Table 5 indicated that there was a significant indirect effect via social exchange with 95% bias-corrected confidence intervals [0.08, 0.26] based on 5,000 bootstrapped samples. Therefore, Hypothesis 4 is supported.

**TABLE 5:** Bootstrapping estimates for mediation and moderated mediation.

Mediation					
			95% bias-corrected CI		
Path	Indirect effect		*SE*	LLCI	ULCI
EL→SC→TC	0.17		0.04	0.08	0.26

**Moderated mediation**					

			**95% bias-corrected CI**		
**Dependent variable**	**Level of power distance**	**Effect**	***SE***	**LLCI**	**ULCI**

Subordinate taking charge	Low (−1 SD)	0.31	0.05	0.21	0.41
	High (+ 1 SD)	0.05	0.04	−0.04	0.13

**FIGURE 2:**
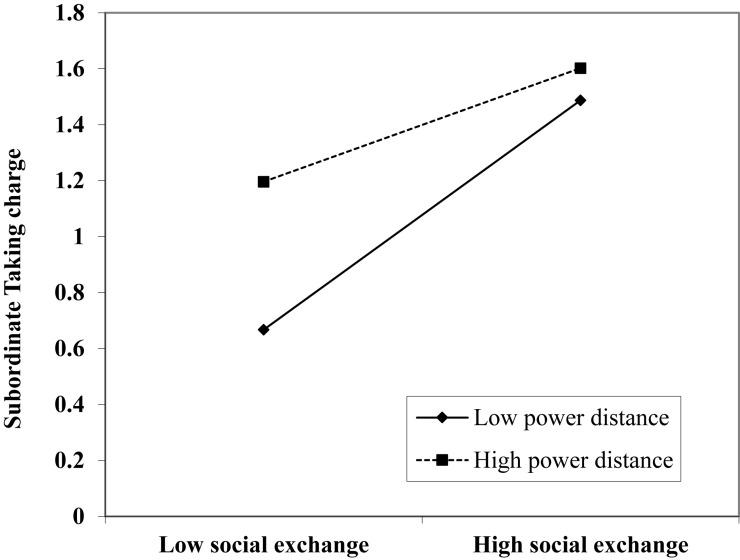
Interaction plot of social exchange and power distance on taking charge.

For Hypothesis 5, the interactive effect of social exchange and power distance on subordinate taking charge was also significant (β = −0.21, *p* < 0.001, Model 6). Following the procedures recommended by Dawson (2014), we drew an interaction plot, which is shown in Figure 2. The results of a simple slope test indicate that the influence of social exchange on subordinate taking charge was more positive and prominent with a low (*b* = 0.41, *p* < 0.001) rather than high (*b* = 0.20, ns) level of social exchange. Therefore, Hypothesis 5 is supported.

Hypothesis 6 predicts that power distance moderates the indirect influence of ethical leadership on subordinate taking charge via social exchange. We also used the PROCESS macro (Hayes, 2013), which offers an overall index of the moderated mediation to test the differences of indirect effects at high (1 SD above the mean) and low (1 SD below the mean) levels of the moderator. Table 5 shows that the indirect influence of ethical leadership on taking charge through social exchange was significant when power distance was low (*b* = 0.31, 95% CI = [0.21, 0.41]). However, the indirect effect became insignificant with high power distance (*b* = 0.05, 95% CI = [−0.04, 0.13]). Therefore, Hypothesis 6 is supported.

## Discussion

Our research examined how and when ethical leadership facilitates subordinate taking charge. We tested that ethical leadership promoted subordinate taking charge theoretically and empirically. Drawing on social exchange theory, we confirmed that social exchange mediates the relationship between ethical leadership and subordinate taking charge. Moreover, power distance moderated the influence of social exchange on subordinate taking charge. Social exchange was positively related to subordinate taking charge only for subordinates with low power distance. Finally, the results showed that the moderated mediation association between ethical leadership and subordinate taking charge via power distance was stronger under low power distance.

### Theoretical Implications

There are three theoretical contributions of our research to the existing literature on ethical leadership and subordinate taking charge. Firstly, our research facilitates the understanding that ethical leadership is a motivating effect on subordinate taking charge. As mentioned above, previous research about ethical leadership has verified its influence on different proactive behaviors, such as voice (Zhu et al., 2015; Hu et al., 2018) and organizational citizenship behavior (Mo and Shi, 2017; Shareef and Atan, 2019). Previous researches relate to taking charge as a prosocial and discretionary behavior of enhancing management effectiveness (Morrison and Phelps, 1999; Grant et al., 2009; Lee, 2016). Nevertheless, the effect of ethical leadership on subordinate taking charge has not been thoroughly discussed (Lee, 2016). Our research provides empirical evidence of the beneficial effects of ethical leadership, specifically on subordinate taking charge. Additionally, in the study of Lee (2016), participants were soldiers of the South Korean Navy, but our research is the first to examine the relationship between ethical leadership and subordinate taking charge in real work contexts.

Secondly, our study found social exchange to be a critical mediating mechanism in the ethical leadership–subordinate taking charge relationship. On the basis of social exchange theory, ethical leadership could promote subordinates’ social exchange, which would promote subordinate taking charge in turn. Generally, our results indicate the potential benefits of ethical leadership and that its effect on subordinate taking charge is exerted via social exchange. Meanwhile, this research empirically tests previous research that social exchange is helpful in explaining that ethical leadership affects subordinate behavior (Brown and Treviño, 2006) and responds to calls for “focus on the general form of the exchange relationship” (Shore et al., 2006).

Thirdly, our results shed light on the indirect relationship between ethical leadership and subordinate taking charge through social exchange conditional on power distance. Social exchange has a greater effect on subordinate taking charge when the subordinate has a low power distance. Therefore, another contribution of this study is the identification of the contextual boundary conditions that shape the nature of the ethical leadership–subordinate taking charge relation. Specifically, our research not only theoretically identified the interaction effect of social exchange and power distance on subordinate taking charge but also empirically examined the moderating role of power distance in the relationship between social exchange and subordinate taking charge.

### Practical Implications

Our findings also provide some practical suggestions. First, organizations should inspire managers to show high standards of ethics, show more concern about their subordinates, and establish and improve rewards of work-related behavior. It is worthwhile to make such efforts because they could promote the development of subordinates’ social exchange; subordinates with a high level of social exchange are more likely to take part in taking charge. Besides, organizations should hire leaders who have a higher ethical level, reward and evaluate ethical behaviors in leadership work, and foster ethical leadership through ethical training programs.

Second, this study shows that social exchange plays a key mediating role in the relationship between ethical leadership and subordinate taking charge. Supervisors ought to take steps to enhance subordinates’ social exchange, which is dynamic and could be promoted through leader relations (Shore et al., 2006). For example, supervisors should integrate relevant management practices and increase the chance of private communication to foster high-quality social exchange, which can promote subordinate taking charge in turn.

Third, our study indicates that power distance plays an important role in predicting the degree to which subordinates may have the intent of taking charge. Supervisors should notice subordinates who have lower power distance, develop high-quality social exchange relationships, and promote taking charge by paying more attention to subordinates, such as showing concern for their daily work and life. Thus, to facilitate subordinate taking charge, managers need to pay attention to subordinate’s power distance manifested in daily interactions.

### Limitations and Future Research

Our study has several limitations. First, this research has a cross-sectional research design, which cannot eliminate the possibility of reciprocal or reverse relationships and prevents us from making strong causal relationships. For example, social exchange could engender subordinate perceptions of ethical leadership, and taking charge could be attributed to social exchange, to reverse the direction of causality. Future researches should use a longitudinal research design to explore potentially reciprocal relationships and clarify the direction of causality.

Second, our study only collects samples of work teams from Chinese firms, so there may exist the problem of generalizability, which is probably not suitable for other countries, cultures, and organizations. Therefore, we hope that future studies can replicate our research to different countries, different cultural backgrounds, and different organizational sections to overcome this limitation.

Third, our study finds that social exchange has a mediating role in the relationship between ethical leadership and subordinate taking charge. However, other mediating mechanisms may explain ethical leadership on subordinate taking charge, and future research could examine some psychological variables which mediate this relationship, for instance, intrinsic motivation, psychological safety, and organizational identification. Moreover, future research can explore the mediating mechanism between social exchange and subordinate taking charge. Subordinates’ positive emotions, such as the feelings of being inspired, enthusiastic, or proud, as the mediating variables between social exchange and taking charge can be used. The kind of exploration can further clarify the mechanisms by which social exchange affects subordinate taking charge.

Finally, our study indicates that power distance has a moderating effect on the relationship between social exchange and subordinate taking charge. Future studies could examine other possible boundary conditions of different personal characteristics. For instance, empirical work could explore whether factors such as entity morality beliefs, risk aversion, or political skill strengthen or suppress the effects of ethical leadership on social exchange and subordinate taking charge.

## Data Availability Statement

The datasets generated for this study are available on request to the corresponding author.

## Ethics Statement

The studies involving human participants were reviewed and approved by the School of Economics and Management at Nanjing University of Science and Technology. The patients/participants provided their written informed consent to participate in this study.

## Author Contributions

QW and JB designed the study. QW get the data and wrote the manuscript. XiZ, XuZ, and WJ revised the manuscript.

## Conflict of Interest

The authors declare that the research was conducted in the absence of any commercial or financial relationships that could be construed as a potential conflict of interest.
